# Runx2 Suppresses Astrocyte Activation and Astroglial Scar Formation After Spinal Cord Injury in Mice

**DOI:** 10.1007/s12035-024-04212-6

**Published:** 2024-05-25

**Authors:** Leilei Lu, Jiazong Ye, Dafa Yi, Tengfei Qi, Tong Luo, Silei Wu, Liangliang Yang, Lei Li, Hongyu Zhang, Daqing Chen

**Affiliations:** 1 Department of Emergency, The Second Affiliated Hospital and Yuying Children’s Hospital, Wenzhou Medical University, Wenzhou, Zhejiang 325027 China; 2 School of Pharmaceutical Sciences, Wenzhou Medical University, Wenzhou, Zhejiang 325000 China; 3 School of Pharmaceutical Sciences, Cixi Biomedical Research Institute, Wenzhou Medical University, Wenzhou, Zhejiang 325035 China; 4 Department of Ultrasound, Dongtou District People’s Hospital, Wenzhou, Zhejiang 325700 China; 5 The Wenzhou Third Clinical Institute Affiliated To Wenzhou Medical University, Wenzhou Medical University, Wenzhou, Zhejiang 325000 China

**Keywords:** Runx2, Astrocytes, Astroglial scar, Nuclear-matrix-targeting signal, Spinal cord injury

## Abstract

After spinal cord injury, astrocytes undergo a reactive process and form an astroglial scar, which impedes the regeneration of axons. The role of Runx2 in promoting the transformation of astrocytes in the central nervous system is well-established. However, it remains unclear whether Runx2 also plays a role in the development of astroglial scar, and the precise underlying mechanism has yet to be identified. Recently, our study using cell culture and animal models has demonstrated that Runx2 actually suppresses astrocyte activation and the formation of astroglial scar following injury. The initial results demonstrated an increase in the expression of Runx2 in astrocytes following in vivo injury. Subsequently, the overexpression of Runx2 resulted in the inhibition of astrocyte activation, reduction in the total area of astroglial scar, and restoration of neural function after 14 days of injury. However, these effects were reversed by CADD522. These findings indicate that Runx2 could potentially serve as a therapeutic intervention for spinal cord injury (SCI). Furthermore, our findings suggest that the Nuclear-matrix-targeting signal (NMTS) of Runx2 is associated with its effect. In summary, the study’s results propose that targeting Runx2 may be a promising treatment approach for reactive astrocytes and astroglial scar in the recovery of SCI.

**Graphical Abstract:**

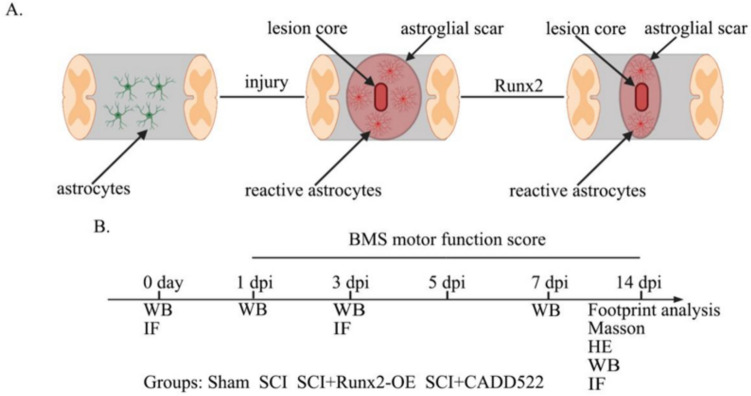

## Introduction

According to estimates, there are over 3 million people worldwide living with spinal cord injuries (SCI) [1]. These injuries can result in severe motor, sensory, and autonomic impairments [2, 3]. Although current therapeutic strategies aim to minimize tissue damage and enhance patients’ quality of life, they do not address the reconstruction of disrupted neuroanatomical circuits. This emphasizes the difficulties researchers encounter in achieving axonal reconnection and neural regeneration in cases of SCI. Astroglial scars, also known as fibrous scars, are scars caused by the activation of astrocytes after spinal cord injury. It is considered a dense restrictive rim surrounding the lesion core and has been identified as a major factor in the failure of spinal cord regeneration [4]. In recent decades, astrocytes have been observed to play important roles in various biological activities in the central nervous system, such as blood-brain barrier regulation, synaptic function, and glutamate uptake [5–7]. In addition, it has an important impact on pathological processes such as neurodegenerative diseases, stroke, and central nervous system damage [8–10]. After spinal cord injury, astrocytes undergo significant transformation, leading to the upregulation of multiple genes, resulting in the formation of astrocytic scars [11]. The function of these reactive astrocytes is controversial, with previous studies reporting central nervous system (CNS) injury and support [12].

The Runt-related transcription factor (Runx) family includes three related transcription factors: Runx1, Runx2, and Runx3 [13]. These genes are defined by a highly conserved 128 amino acid domain called a dwarf homeodomain. During endochondral osteogenesis, Runx2 plays an important role in regulating chondrocyte proliferation, differentiation, and hypertrophy [14]. Previous studies have shown that Runx2 can promote apoptosis of central nervous system (CNS) neurons. Studies have shown that Runx2 in the central nervous system (CNS) can promote the conversion of early astrocytes (Ea) to late astrocytes (IA) during neural progenitor cell (NPC) differentiation [15, 16]. In addition, other studies have also detected Runx2 expression in human gliomas. These results indicate a close connection between Runx2 and astrocytes. A large number of studies have shown that reactive astrocytes play a key role in central nervous system injury [17–19]. However, there is a lack of research on the mechanisms that hinder astrocyte activation in the injured spinal cord.

Therefore, the purpose of this study was to explore the relationship between Runx2 expression and astrocyte activation after spinal cord injury. Astrocytes are rapidly activated after central nervous system injury. Upregulation of Runx2 expression after acute injury may lead to reduced astrocyte reactivity and astrocytic scar formation. This process ultimately leads to functional recovery after injury.

## Materials and Methods

### Animals

Adult female C57BL/6 (20–22 g, *n* = 70) were provided by the Shanghai Zoological Center, Chinese Academy of Sciences. We housed all mice in individually ventilated cages (IVC, 5 per cage) with controlled ambient temperature and light/dark cycle, and had free access to food and water.

### Spinal Cord Injury Model

A mouse model of frustrated spinal cord injury was created as previously described [20]. Briefly, the animals were anesthetized with an overdose of inhaled isoflurane (4%) and maintained in an isoflurane vaporizer with inhaled isoflurane (2%), and the T10 vertebra A laminectomy was performed. After complete exposure of the spinal cord, a contusion was applied to the spinal cord from a height of 50 mm using a MASCIS impactor (WM Keck Collaborative Neuroscience Center, Rutgers, State University of New Jersey). Mice were randomly divided into four groups: Sham group, SCI group, SCI + Runx2-OE (AAV-Runx2) group, and SCI + CADD522 group. There were 15 animals in each group, and 10 animals died due to injuries. After surgery, the bladder was manually emptied three times a day until the bladder reflexes recovered. All animal experimental protocols were approved by the Animal Care and Use Committee of Wenzhou Medical University.

### Cell Culture and Treatment

PC12, BV2, and SVG-P12 were cultured in RPMI1640 medium and DMEM medium containing 10% fetal bovine serum (FBS) and 1% penicillin/streptomycin mixture, respectively, at 37 °C in a humidified atmosphere containing 5% CO_2_. Purchase cells and all reagents discover the potential mechanism of Runx2 inhibiting astrocyte activation in vitro, establish a TBHP-induced cell model, and simulate the microenvironment after spinal cord injury.

### Transduction

Cells were added to a 12-well plate at a concentration of 1 × 10^5 cells/well, reduced with Runx2 small spacer RNA (siRNA-Runx2), and transfected with plasmid serum medium carrying Runx2 gene (Gibco, USA). Lipofectamine 3000 (Thermo Fisher Scientific, USA) was added to improve transfection efficiency. After 6 h, we replaced the medium with a new complete medium.

### Western Blot Analysis

Tissues and cells were lysed with RIPA lysis buffer supplemented with a 1% protein phosphatase inhibitor cocktail and centrifuged (12,000 rpm) at 4 ℃ for 15 min to obtain protein supernatants. Load 40–60 µg of total protein onto a 10% or 12% SDS-PAGE gel and transfer the separated proteins to a PVDF membrane in a transfer buffer. The membrane was then blocked with 5% skim milk for 1 h at room temperature. Then incubate overnight at 4 °C with the following primary antibodies: Runx2 (1:1000; Abcam); GFAP (1:1000; Abcam); polyclonal GAPDH antibody (1:1000; Proteintech); polyclonal S100A10 antibody (1:1000; Proteintech); Neurocan (1:1000; Zenbio); and NF-κB p65 (1:1000; Proteintech). Then, after washing three times with TBST, the membrane was immersed in the corresponding HRP-labeled secondary antibody for 1 h at room temperature. The relative density of the bands was quantitatively analyzed using ImageJ software.

### Histology and Immunofluorescence

We fixed mouse spinal cord tissue sections and cells in 4% PFA for 30 min and blocked with 5% bovine serum albumin (Beyotime) for 1 h. Tissue sections and cells were incubated overnight at 4 ℃ with primary antibodies: NeuN (1:500; Abcam); Iba-1 (1:500; Abcam); GFAP (1:500; Abcam); Runx2 (1:500; Abcam); S100A10 (1:500; Proteintech); and NF-κB p65 (1:500; Proteintech). After washing three times with PBST, primary and secondary antibodies were detected for 1 h at room temperature. For histopathological examination, tissues were stained with a Masson trichrome staining kit and hematoxylin-eosin/HE staining kit (Solarbio) according to the manufacturer’s instructions.

### Functional Behavior Evaluation

Motor recovery was assessed using footprint analysis at 14 dpi. Briefly, the analysis was performed by dipping the animal’s hind paws in red dye and their front paws in blue dye. The animal is then allowed to move through a narrow area. We scanned the footprints and analyzed the resulting images.

The Basso Mouse Scale (BMS) assessment method was used to detect changes in the hind limb motor function of mice after spinal cord injury. All animals were evaluated by two observers blinded to experimental conditions and scored according to a scoring system (hindlimb mobility, coordination, paw posture, trunk stability, and tail posture) as previously described. The rating is from 0 to 9. The outcome measures were determined by three independent assessors.

### Statistical Analysis

The results are expressed as means ± SD of at least three independent experiments, all determined by the experimenter without knowledge of the exact experimental group. For statistical analysis, we used the GraphPad Prism 9 software (GraphPad Software, CA). For simple two-group comparisons, we use an unpaired *t*-test to calculate *p*-values and one-way ANOVA to calculate *p*-values for factor comparisons of three or more groups. If *p* < 0.05, the difference is considered existing.

## Results

### Runx2 Expression Increased After SCI and was Related with Astrocytes

**Fig. 1:**
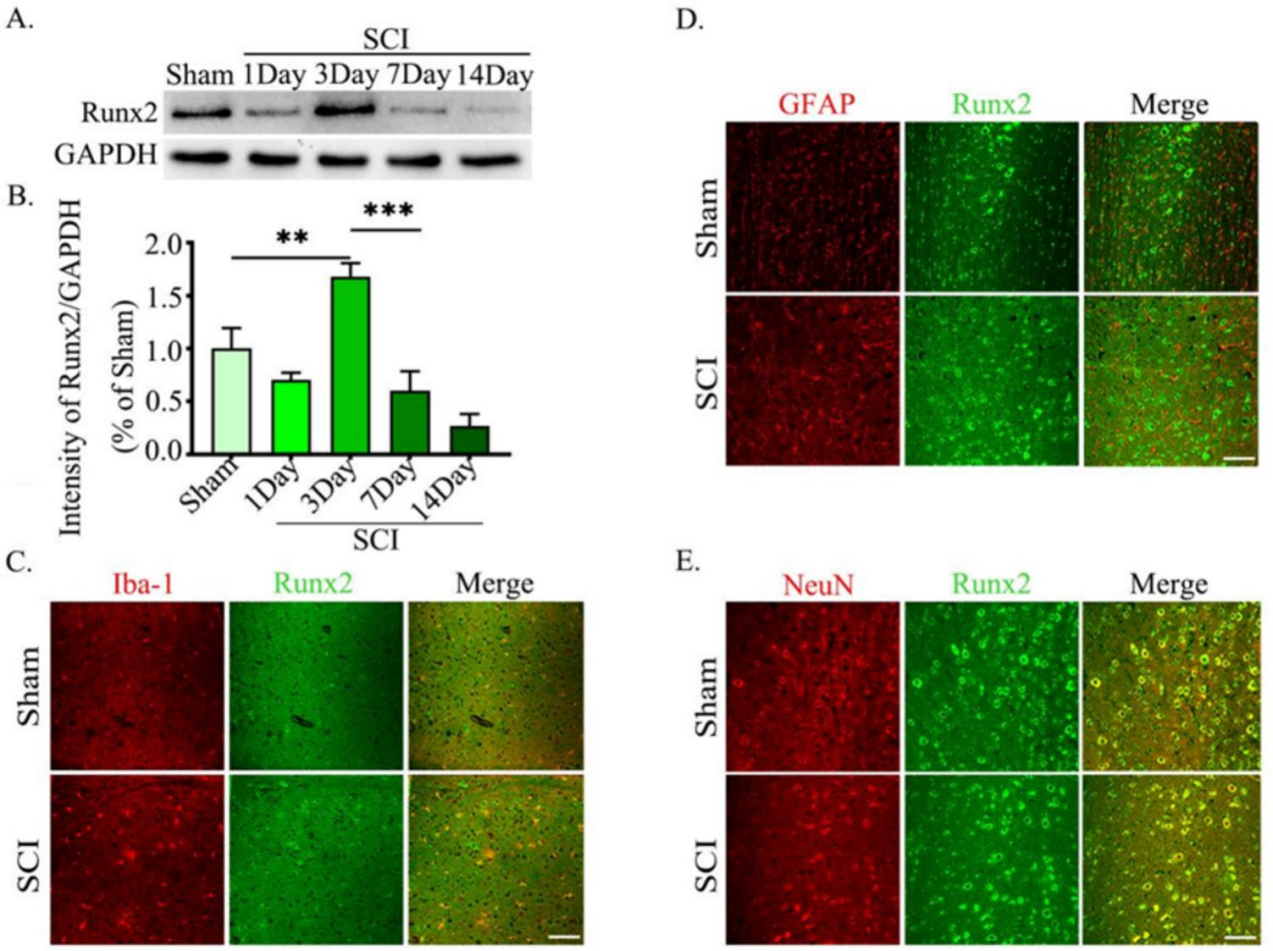
Runx2 expression increased after SCI and was related with astrocytes. **A** Western blot analysis of Runx2 (57 kDa) in lesions, with GAPDH (37 kDa) as an internal loading control. **B** Quantification of result in panel (**A**). The data were analyzed using one-way analysis of variance and all data are expressed as the mean ± S.D. **C–E** Immunofluorescence staining of Runx2 (green) and NeuN, GFAP, or Iba-1（red）in the spinal cord at 3 days post-injury. *p < 0.05; **p < 0.01; ***p < 0.001. Scale bar = 200 μm

To investigate the role of Runx2 in the pathogenesis of SCI, we analyzed the expression of Runx2 in SCI mice and compared it with sham-operated mice. Western blot results showed that compared with sham-operated mice, the expression of Runx2 in the spinal cord of spinal cord injury mice was up-regulated and reached a peak on day 3 (Fig. 1A, B). Therefore, we used tissue from 3 days after spinal cord injury for immunofluorescence detection. To further identify the possible cellular origins and target cells of Runx2 in the spinal cord, we labeled them with antibodies specific for astroglial fibrillary acidic protein (GFAP; astrogliogenic cells), neuronal nuclei (NeuN; neuronal marker) tissue sections around the injury, and ionized calcium-binding adapter molecule-1 (Iba-1; microglia marker). Immunofluorescence results showed that Runx2 was expressed in neurons, astrocytes, and microglia (Fig. 1C-E). Increases in Runx2 were associated only with astrocytes. The above data suggest that Runx2 expression changes after spinal cord injury and is related to astrocytes.

### Overexpressing Runx2 Inhibited the Activation of Astrocytes Following SCI

**Fig. 2:**
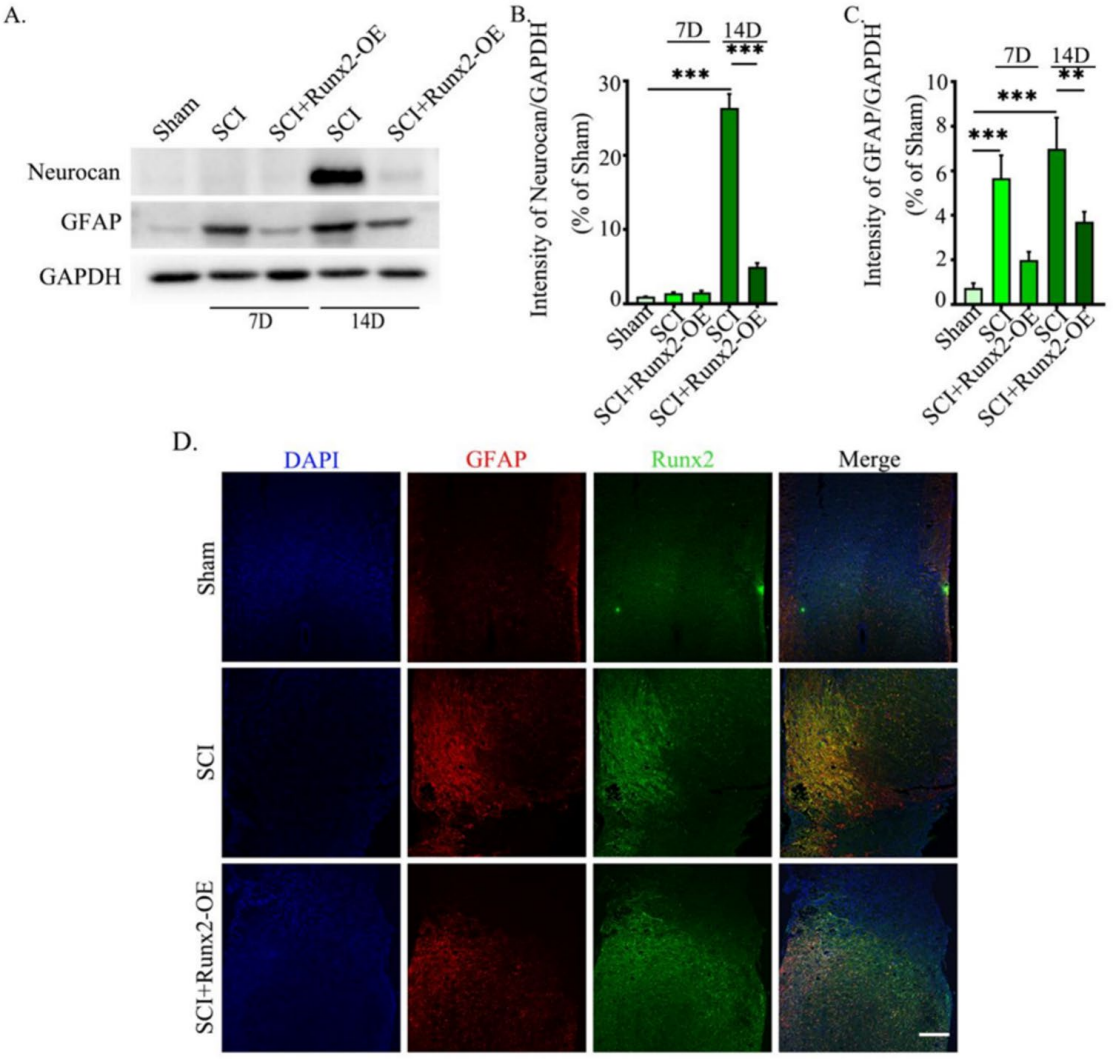
Overexpressing Runx2 inhibited the activation of astrocytes following SCI in vivo. **A** Western blot analysis of Neurocan (143 kDa) and GFAP (55 kDa)in lesions, with GAPDH (37 kDa) as an internal loading control. **B**, **C** Quantification of result in panel (**A**). The data were analyzed using one-way analysis of variance and all data are expressed as the mean ± S.D. **D** Immunofluorescence staining of DAPI (blue), GFAP (red), and Runx2 (green) in the spinal cord at 14 days post-injury. *p < 0.05; **p < 0.01; ***p < 0.001. Scale bar = 200 μm. Runx2-OE (AAV-Runx2)

To further study the role of Runx2 gene after spinal cord injury, we used an adenovirus expression system to overexpress the Runx2 gene. Temporal expression of GFAP and Neurocan was measured on days 7 and 14 after spinal cord injury. After central nervous system injury, Neurocan expression is increased and axonal growth is inhibited. Western blot showed that neurocan expression peaked at 14 dpi, but after overexpression of Runx2, the expression decreased significantly (Fig. 2A, B). Furthermore, GFAP expression in spinal cord injury (SCI) was significantly increased at 7 and 14 dpi compared with the sham group, while Runx2-oe (AAV-Runx2) treatment reduced GFAP expression in SCI (Fig. 2A, C). Therefore, the effect of Runx2 was analyzed using 14 days after Sci as a control point. In immunofluorescence experiments, we observed that the expression of GFAP in SCI was higher than that in the sham group and was reversed by Runx2-OE (AAV-Runx2) (Fig. 2D). Taken together, these results suggest that increases in Runx2 may reduce astrocyte activation.

### Overexpressing Runx2 Improved the Locomotor Function by Reducing the Formation of Astroglial Scar

**Fig. 3:**
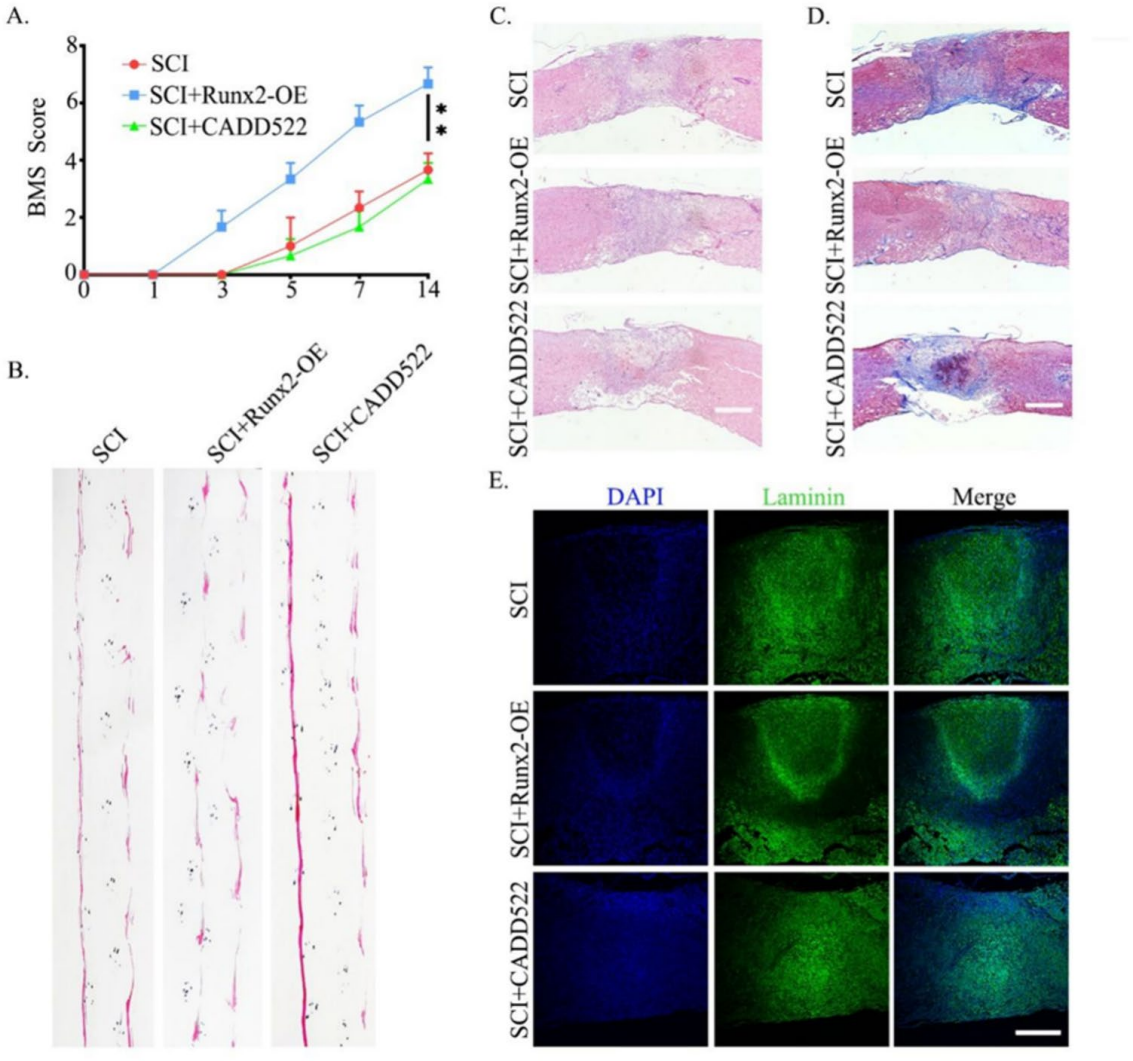
Overexpressing Runx2 improved the locomotor function by reducing the formation of astroglial scar. **A** Statistical analysis of BMS scores of mice from different groups at the indicated time point after SCI. The data were analyzed using one-way analysis of variance and all data are expressed as the mean ± S.D. **B** Footprint test of mice in different groups at 14 days post-injury. **C**, **D** Assessment of astroglial scar by HE and MASSON at 14 days after SCI. **E** Immunofluorescence staining of DAPI (blue) and Laminin (green) in the spinal cord at 14 days post-injury. *p < 0.05; **p < 0.01, ***p < 0.001. Scale bar = 200 μm

In order to evaluate the beneficial effect of Runx2 on improving the motor function of mice with spinal cord injury, the Basso Mouse Scale (BMS) scoring method and the footprint method were used to evaluate the motor function of mice after sham surgery and AAV-Runx2 treatment. Fourteen days after spinal cord injury, the average BMS score and footprint of the AAV-Runx2 treatment group were higher than those of the sham operation group, indicating that AAV-Runx2 treatment significantly improved motor function (Fig. 3A, B). Similarly, the results of HE and MASSON also showed that the astrocyte scar area was reduced after Runx2 overexpression (Fig. 3C, D). In immunofluorescence experiments, we observed a significant decrease in laminin levels in the AAV-Runx2 treated group compared with the simple disruption group (Fig. 3E).

To further investigate the role of Runx2 in astrocyte scar formation, we injected experimental animals with CADD522 (an antagonist that binds to Runx2 DNA) at a concentration of 20 mg/kg via intraperitoneal injection until the day before execution. The results showed that the changes observed in the AAV-Runx2 group were offset by CADD522 (Fig. 3A–E). The above results indicate that overexpression of Runx2 contributes to the recovery of motor function after spinal cord injury by reducing astrocyte scar formation.

### The Expression of Runx2 in Nucleus Increased in SVG-P12 After Injury

**Fig. 4:**
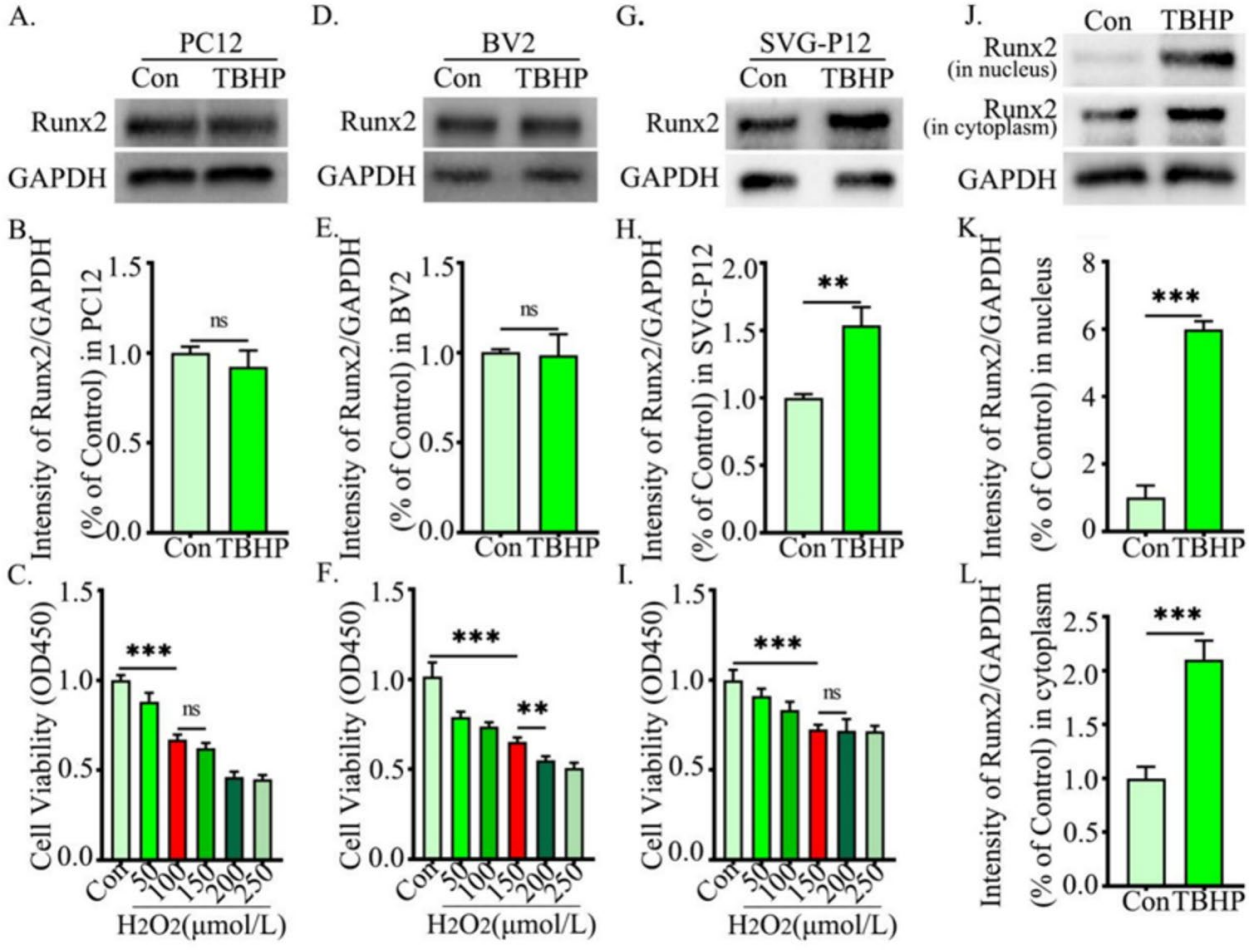
The expression of Runx2 in nucleus increased in SVG-P12 after injury. **A** Western blot analysis of Runx2 (57 kDa) in PC12, with GAPDH (37 kDa) as an internal loading control. **B** Quantification of result in panel (**A**). **D** Western blot analysis of Runx2 (57 kDa) in BV2, with GAPDH (37 kDa) as an internal loading control. **E** Quantification of result in panel (**D**). **G** Western blot analysis of Runx2 (57 kDa) in SVG-P12, with GAPDH (37 kDa) as an internal loading control. **H** Quantification of result in panel (**G**). **J** Western blot analysis of Runx2 (57 kDa) in nucleus and cytoplasm in SVG-P12, with GAPDH (37 kDa) as an internal loading control. **K**, **L** Quantification of result in panel (**J**). The data were analyzed by two-tailed unpaired Student’s *t*-test, and the results represent as the mean ± S.D. **C**, **F**, **I** CCK8 assay was performed for the most suitable concentration in PC12 (**C**), BV2 (**F**), and SVG-P12 (**I**). The data were analyzed using one-way analysis of variance, and all data are expressed as the mean ± S.D. *p < 0.05; **p < 0.01; ***p < 0.001

To confirm the expression of Runx2 in astrocytes, we used TBHP to simulate the microenvironment of local tissues after injury. We first evaluated the cytotoxicity of TBHP to determine the appropriate concentration for each cell type (Fig. 4C, E, I). Subsequently, we observed a significant increase in Runx2 expression in SVG-P12 (Fig. 4G, H), while no obvious changes were observed in other cells (Fig. 4A, B, D, E).

To further study the changes of Runx2 in SVG-P12, we detected the expression levels of Runx2 in the nucleus and cytoplasm. Western blot results showed that compared with the control group, protein expression in the nucleus changed significantly after stimulation (Fig. 4J–L). In conclusion, the expression of Runx2 in the nucleus increased significantly after SVG-P12 injury.

### The Effect of Runx2 Nuclear Import is Crucial on Modulation of Astroglial Scar

**Fig. 5:**
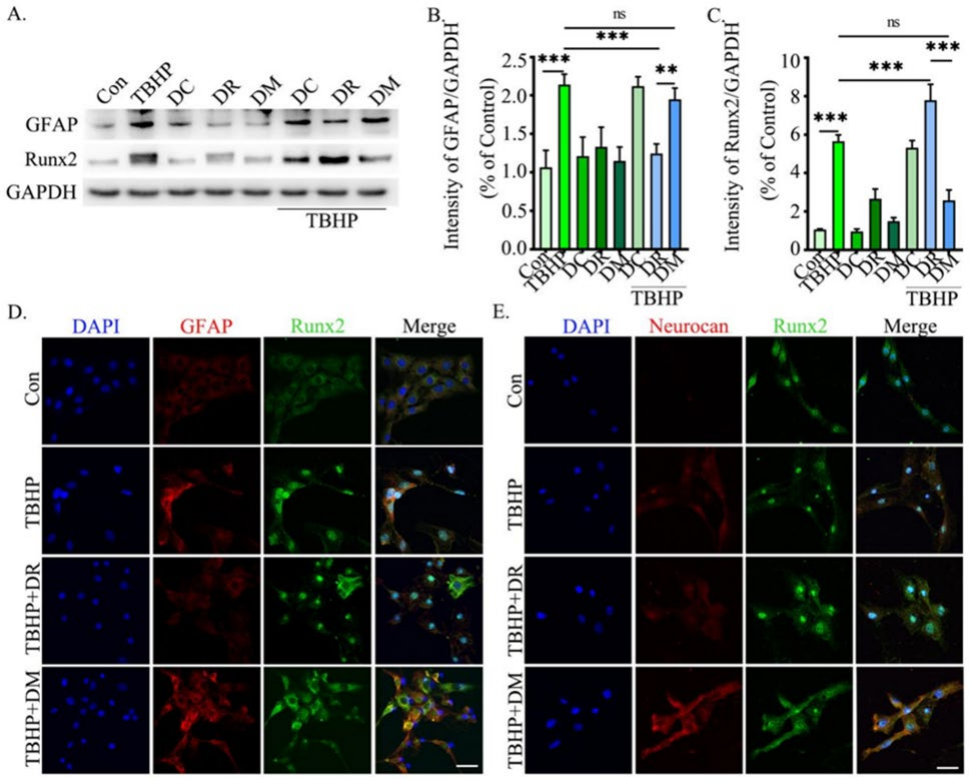
The effect of Runx2 nuclear import is crucial on modulation of astroglial scar. **A** Western blot analysis of Runx2 in nucleus (57 kDa) and GFAP (55 kDa), with GAPDH (37 kDa) as an internal loading control. **B**, **C** Quantification of result in panel (**A**). **D** Immunofluorescence staining of DAPI (blue), GFAP (red), and Runx2 (green) in the SVG-P12. **E** Immunofluorescence staining of DAPI (blue), Neurocan (red), and Runx2 (green) in the SVG-P12. The data were analyzed using one‑way analysis of variance, and all data are expressed as the mean ± S.D. *p < 0.05; **p < 0.01; ***p < 0.001. Scale bar = 20 μm. DC (plasmid without Runx2), DR (plasmid of Runx2 with NMTS), DM (plasmid of Runx2 without NMTS)

To investigate the role of Runx2 nuclear import in astrocyte scarring, we analyzed the expression of GFAP and Runx2 in SVG-P12 cells. Western blot analysis showed that the expression of Runx2 protein in the nucleus of cells treated with DR (containing Runx2 plasmid of NMTS) significantly increased, while there was no difference in the expression of Runx2 protein in the nucleus of cells treated with DM (containing Runx2 plasmid without NMTS) (Fig. 5A, C). Furthermore, as Runx2 expression increased in the nucleus, GFAP protein expression decreased. However, there was no significant difference in GFAP between the TBHP group and the TBHP + DM group (Fig. 5A, B). Likewise, our experimental immunofluorescence results showed that Runx2 in the nucleus, GFAP, and Neurocan was increased after TBHP. However, after DR overexpressed Runx2, the expression levels of GFAP and Neurocan were significantly reduced (Fig. 5D, E). Interestingly, these changes were not detected in cells TBHP + DM-treated group. These findings showed that the import of Runx2 into the nucleus is essential for regulating astrocyte activation and reducing the formation of astroglial scar.

### Knockdown of Runx2 Promoted Astrocyte Activation

**Fig. 6:**
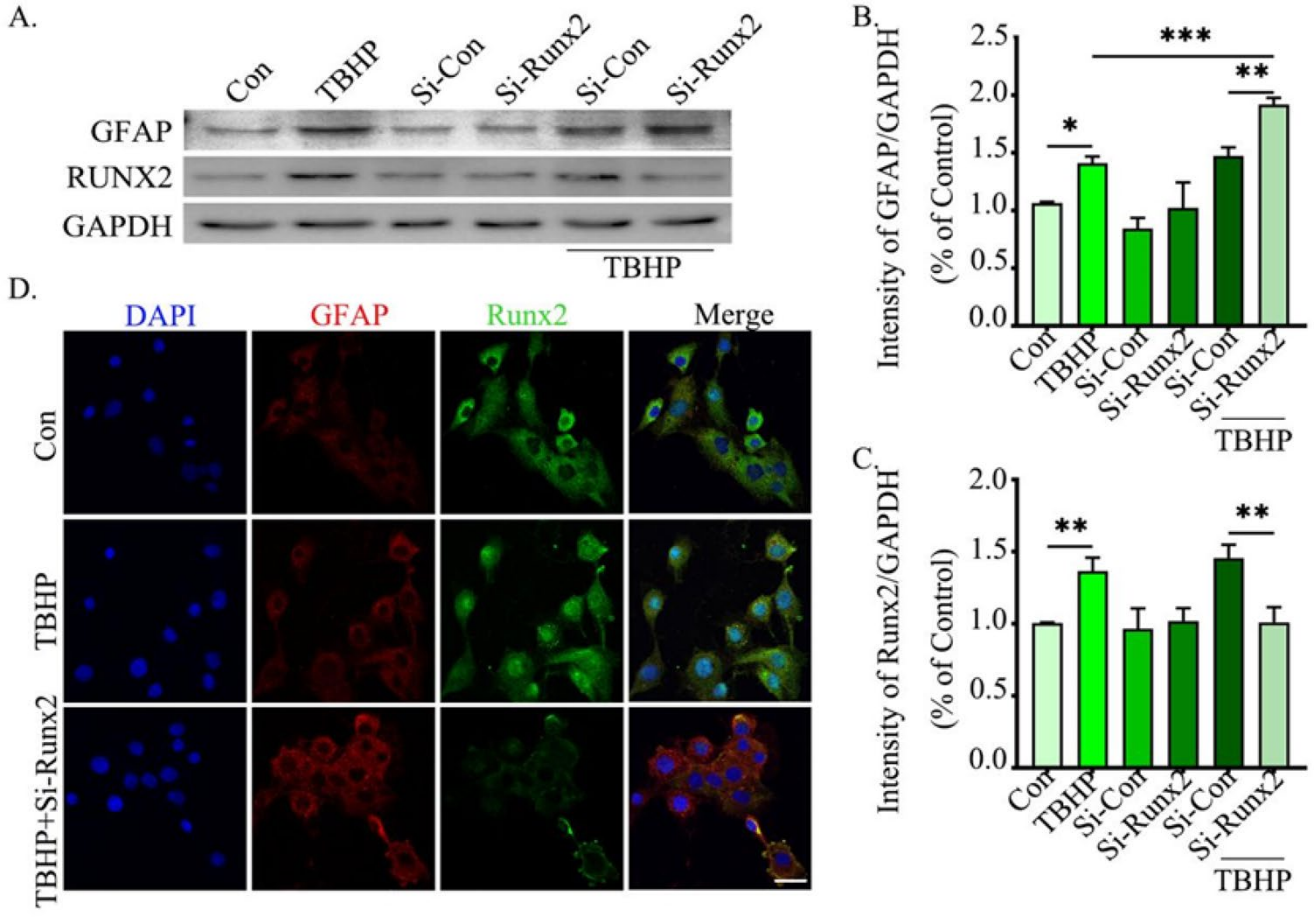
Knockdown of Runx2 promoted astrocytes activation. **A** Western blot analysis of Runx2 in nucleus (57 kDa) and GFAP (55 kDa), with GAPDH (37 kDa) as an internal loading control. **B**, **C** Quantification of result in panel (**A**). **D** Immunofluorescence staining of DAPI (blue), GFAP (red), and Runx2 (green) in the SVG-P12. The data were analyzed using one‑way analysis of variance, and all data are expressed as the mean ± S.D. *p < 0.05; **p < 0.01; ***p < 0.001. Scale bar = 20 μm

To further verify the effect of Runx2 on astrocyte activation, we transfected small spacer RNA into SVG-P12 to disrupt the expression of Runx2. Western blot analysis showed that GFAP protein expression was significantly increased in TBHP-treated cells (Fig. 6A, B). In addition, since si-RNA reduced the expression of Runx2 in the nucleus, the expression level of GFAP protein further increased (Fig. 6A–C). Immunofluorescence results showed that Runx2 and GFAP increased in the nucleus after TBHP. However, knockdown of Runx2 with si-RNA significantly increased the expression of GFAP (Fig. 6D). These results indicate that Runx2 can inhibit astrocyte activation after injury.

## Discussion

Previous studies have shown that astrocytes are activated after spinal cord injury, participate in astrocyte scar formation, and inhibit functional recovery after spinal cord injury [21]. Currently, there are many stimulation methods to inhibit astrocyte activation after injury, including biological materials, protein molecules, and anti-inflammatory drugs [22–25]. However, the presence of the blood-spinal cord barrier or different dosages can improve the efficiency of drug delivery [26, 27]. Current treatment options for astrocyte scars have limitations and shortcomings.

To our knowledge, this study is the first to demonstrate that Runx2 can inhibit astrocyte activation and astrocyte scar formation after SCI in mice and promote the recovery of motor function. In our study, we found that the expression of Runx2 in astrocytes changed significantly after SCI. We then used an adeno-associated virus to overexpress Runx2 in animals and underwent spinal cord injury surgery. When Runx2 was overexpressed in astrocytes using an adeno-associated virus, reduced astrocyte activation was observed after spinal cord injury. Furthermore, immunofluorescence data showed the presence of Runx2-overexpressing astrocytes around the injured tissue, and the size of the astrocytic scar was reduced, which is consistent with the above results.

For transcription factors, the role of the nucleus is critical for their transcriptional regulatory activity. Our study demonstrates that Runx2 has a nucleating role in astrocytes. Although some studies have reported the nuclear localization sequence of Runx2 protein, it is still uncertain whether its effects on the nervous system are attributed to the nucleus [28, 29]. In our study, we observed the nuclear localization of Runx2 in mouse and human astrocytes. To examine the effect of nuclear Runx2 on astrocyte activation, we used plasmids to overexpress Runx2 in astrocytes without the need for nuclear localization sequences. Our results show that increases in nuclear Runx2 lead to significant differences in reactive astrocytes after injury. This differs from approaches that modulate reactive astrocytes by modulating complex signaling pathways [30, 31]. Our study discovered a method to change astrocyte fate by regulating gene expression. Nuclear localization sequences are thought to play an important role in maintaining the function of reactive astrocytes.

Astroglial scar is a unique pathological structure that appears after a central nervous system injury. Although the role of scars after spinal cord injury (SCI) is complex and controversial, the formation of hypertrophic fibrous scars around the injury site and the overexpression of chemical inhibitory factors create an inhibitory environment for regeneration [4, 32]. Astroglial scars are boundaries formed by astrocytes around the injury site, forming inactive areas that hinder axonal regeneration and neurological recovery [4, 33]. Studies have shown that astrocyte activation is often related to many factors, such as LPS and OGD treatment [34, 35]. Anti-inflammatory treatments have been shown to be effective in suppressing changes in spinal cord injury, but it is important to note that inflammation is not the only factor. Other factors such as oxidative stress, apoptosis, and magnetic circuit stimulation also affect astrocyte activation after SCI [36–38]. Compared with these factors, direct intervention of specific transcription factors to reverse astrocyte activation after injury may be a more effective approach.

Similarly, our data show that 14 days after injury in vivo, astrocytes with high GFAP expression accumulate around the lesion site and form an astrocytic scar. Furthermore, an increase in GFAP was observed in SVG-P12 upon TBHP stimulation. Overexpression of Runx2 results in reduced activation of astrocytes, ultimately reducing the likelihood of these cells accumulating around lesions. It can be seen that changing its activation status through Runx2 expression can reduce astrocyte scar formation. Therefore, Runx2 may prevent astrocyte scar formation by inhibiting astrocyte activation.

In conclusion, compared with other published studies, this study is the first to demonstrate that Runx2 inhibits astrocyte activation and astrocytic scar formation after spinal cord injury in mice. This suggests that modulation of Runx2 may be an effective therapeutic target for spinal cord injury. However, the mechanism by which Runx2 regulates astrocyte activation and astrocyte scar formation requires further investigation. Before the clinical implementation of this project, we need to establish a safer and more effective management model. The combined use of biomaterials may prove to be a promising strategy to achieve this goal.

## Data Availability

The original contributions presented in the study are included in the article/supplementary material, further inquiries can be directed to the corresponding authors.
